# The Utility of Point-of-Care Ultrasound for Post-Bronchoscopy Pneumothorax Evaluation

**DOI:** 10.7759/cureus.15339

**Published:** 2021-05-30

**Authors:** Dallis Q Ngo, Ghulam Aftab, Douglas Frenia

**Affiliations:** 1 Pulmonary Medicine, Saint Peter’s University Hospital/Rutgers Robert Wood Johnson Medical School, New Brunswick, USA; 2 Pulmonary Critical Care, Saint Peter’s University Hospital/Rutgers Robert Wood Johnson Medical School, New Brunswick, USA

**Keywords:** pneumothorax ptx, fiberoptic flexible bronchoscopy, electromagnetic navigation, pocus (point of care ultrasound), pulmonary disease, pulmonary critical care, lung biopsy

## Abstract

We present a case of a 65-year-old female with a prior history of B-cell lymphoma with new CT chest findings of a nodule requiring an electromagnetic navigational bronchoscopy with transbronchial biopsies. Post-bronchoscopy, the patient complained of dyspnea and left scapular pain despite two normal anterior-posterior chest X-rays. Point-of-care ultrasound of the lung demonstrated a lack of lung sliding, which was confirmed via a right lateral decubitus chest X-ray. This case illustrates the utility and superiority of lung point-of-care ultrasound while highlighting the limitations of conventional imaging modalities in a post-bronchoscopy evaluation.

## Introduction

The management of pulmonary nodules and lung cancer frequently involves tissue biopsy by bronchoscopy. Although a tissue diagnosis aids tremendously in the proper management of patients, the procedures are not without risks. Pneumothorax is a well-known, yet relatively uncommon, complication of the diagnostic procedure when performed by a skilled operator. Prompt identification of such an adverse event is often made by chest X-ray, however, it has poor sensitivity. We present a case of an individual with an iatrogenic pneumothorax post-bronchoscopy, highlighting the utility and superiority of lung point-of-care ultrasound while demonstrating the limitations of conventional chest X-ray for the evaluation of pneumothoraces in this setting.

## Case presentation

**Figure 1:**
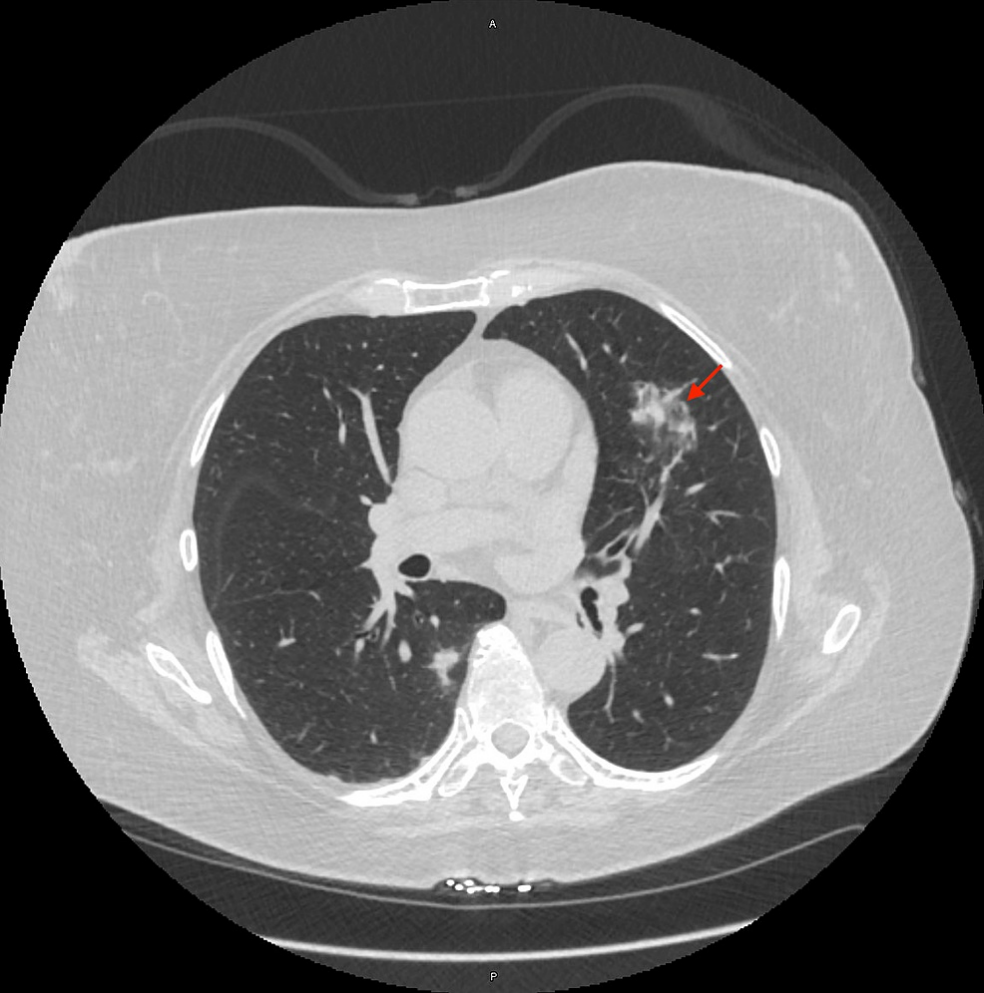
CT chest Red arrow - Superior lingular lesion

A 65-year-old female with a prior history of B-cell lymphoma status post-chemotherapy in 2016 and currently in remission presented to the pulmonary clinic for further evaluation of an abnormal CT chest in comparison to prior scans four months prior. Her new CT scan showed a previously noted spiculated nodule that has changed in configuration, measuring 39 x 11 x 8 mm (Figure 1) as compared to 14 x 12 x 12 mm seen prior. She underwent an electromagnetic navigational bronchoscopy (ENB) with transbronchial biopsies taken from the abnormal lesion localized to the superior segment of the lingula under fluoroscopy guidance. The procedure was completed without procedural complications, and the patient was transferred to the post-anesthesia care unit.

Assessment

**Figure 2:**
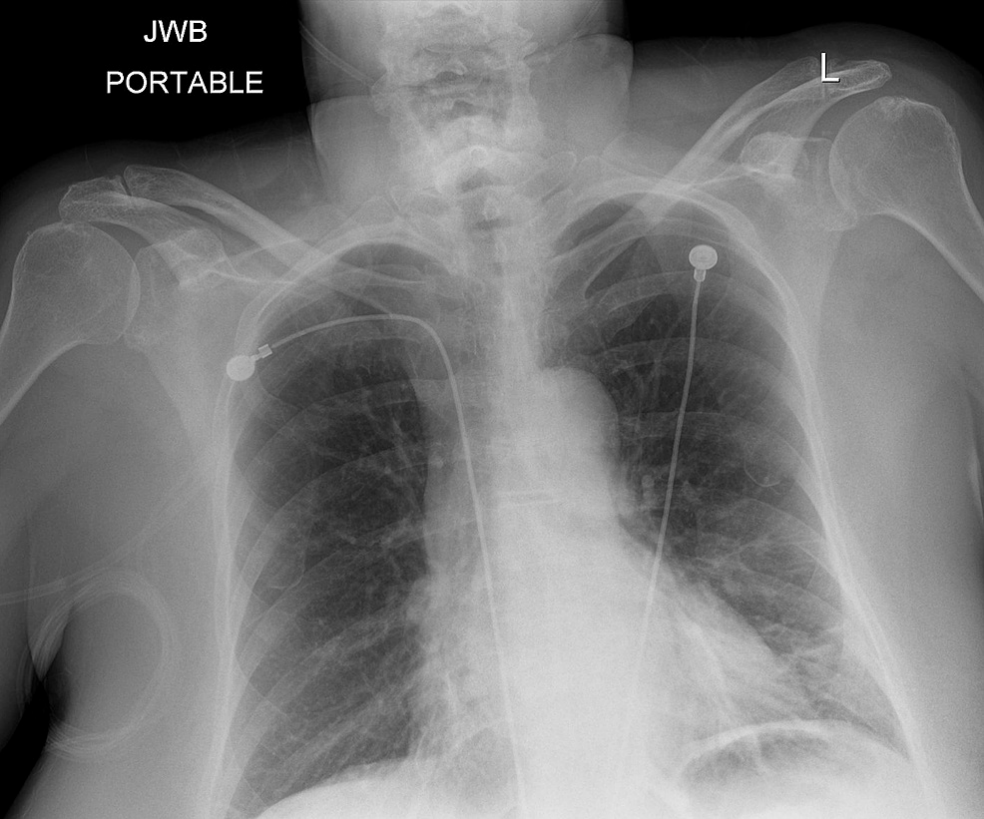
Post-bronchoscopy chest X-ray

**Figure 3:**
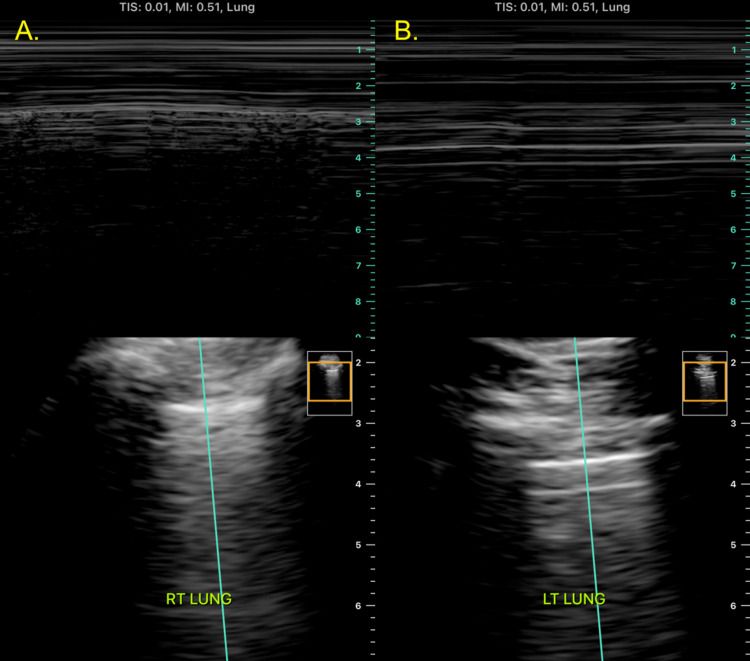
Lung ultrasound: M-mode A. Ultrasound of the right lung apex with M-mode demonstrating a "seashore sign" seen in the presence of lung sliding. B. Ultrasound of the left lung apex with M-mode demonstrating a "barcode/stratosphere sign" seen in the absence of lung sliding. This is suggestive of a pneumothorax.

**Figure 4:**
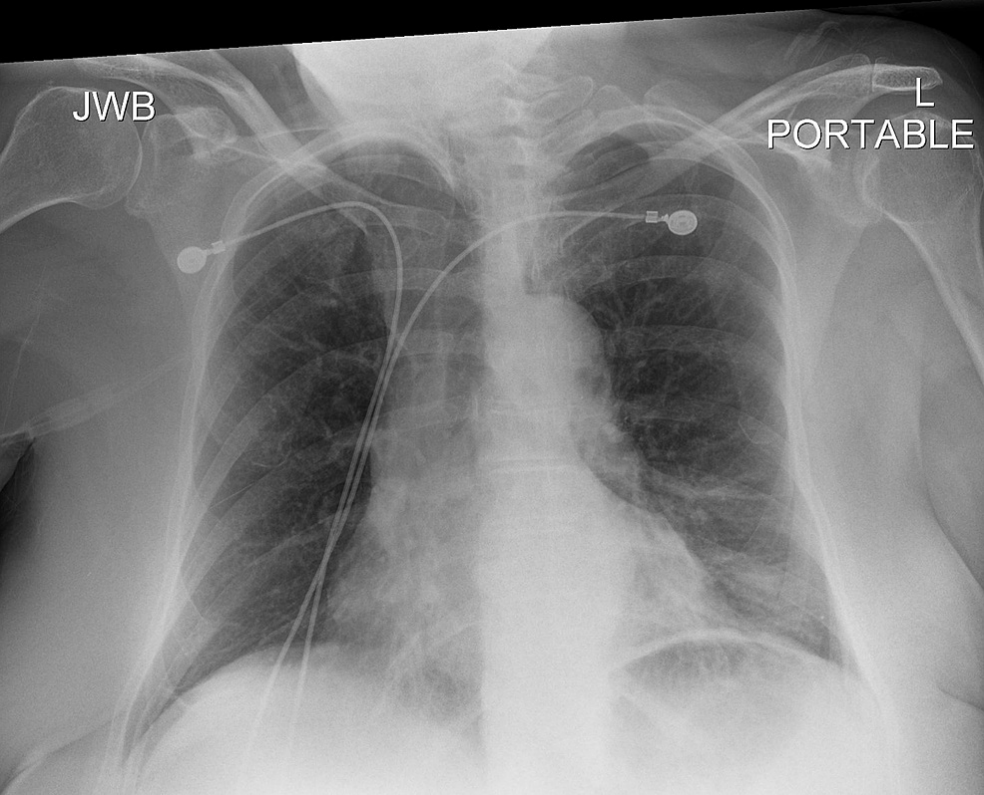
Repeat chest X-ray

**Figure 5:**
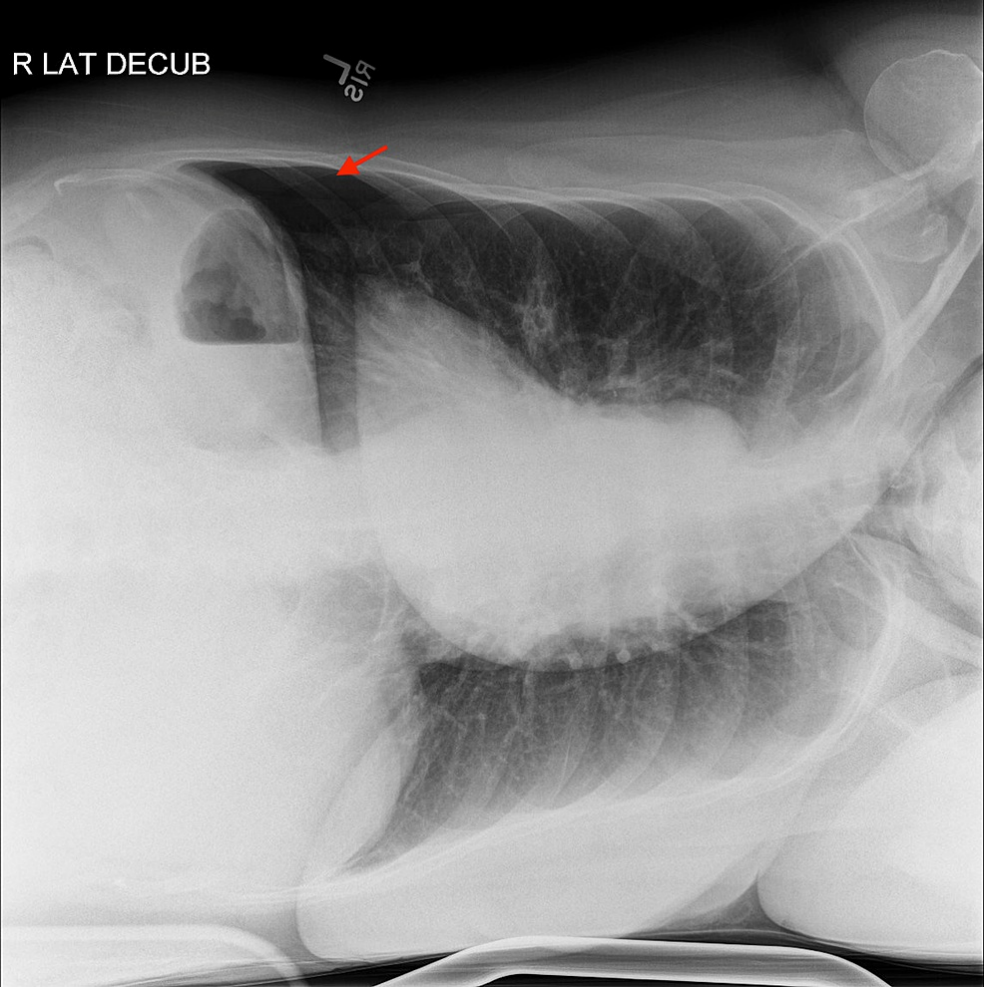
Right lateral decubitus chest X-ray Red arrow - Left pneumothorax

A post-bronchoscopy chest X-ray (CXR) (Figure 2) showed small new opacities in the lingula, expected of the procedure, but no pneumothorax was appreciated. Shortly afterward, the patient started to experience mild dyspnea and left scapular pain, therefore, a bedside point-of-care ultrasound (POCUS) was performed, which revealed normal lung sliding over the right upper lobe but the absence of lung sliding over the left upper lobe. M-mode on ultrasound was utilized, which showed a “seashore sign” over the right upper lobe and a “barcode/stratosphere sign” over the left upper lobe (Figure 3). A repeat CXR did not demonstrate findings of a left pneumothorax (Figure 4); therefore, a right lateral decubitus CXR was performed, which successfully demonstrated radiographic findings of a left pneumothorax (Figure 5).

Diagnosis

The patient was diagnosed with a small iatrogenic left pneumothorax.

Management

Supplemental oxygen was administered via a non-rebreather mask with 15 liters of O_2_ per minute to help accelerate pneumothorax reabsorption, and the patient was admitted overnight for observation. Repeat chest radiograph the following morning revealed a stable left pneumothorax, and the patient’s symptoms of dyspnea and scapular pain had resolved. She was discharged home with instructions to return to the clinic to review her bronchoscopy pathology results.

## Discussion

Transthoracic needle aspiration (TTNA) with CT guidance has historically been a commonly utilized strategy for the diagnosis of cancer in peripheral lung lesions. Although it has a high sensitivity of 81%-97% for a successful diagnosis, TTNA has been associated with pneumothorax in up to 19%-25% of cases [1-2]. Over the past two decades, ENB has emerged as a modality for the evaluation of peripheral pulmonary lesions, which has a lower diagnostic yield of 66% but with the advantage of a lower pneumothorax risk [1]. According to a meta-analysis, ENB was associated with a pneumothorax rate of 1.5% [3].

Although a post-bronchoscopy CXR is commonly performed, a meta-analysis by Alrajab et al. demonstrated that CXR has a sensitivity and specificity of 39.8% and 99.3%, respectively, for the successful diagnosis of pneumothorax [4]. In contrast, lung ultrasound has shown to have superior diagnostic accuracy. A meta-analysis by Ebrahimi et al. revealed a sensitivity and specificity of 87% and 99%, respectively [5]. This case highlights the utility and superiority of lung POCUS for post-bronchoscopy evaluation for pneumothorax and the limitations of conventional imaging modalities, such as CXR, in this setting.

## Conclusions

A pneumothorax is a commonly reported complication post-bronchoscopy, especially when performing multiple transbronchial biopsies. Although a conventional post-bronchoscopy examination commonly includes a chest X-ray, it may occasionally not be successful in identifying a pneumothorax radiographically. Adopting a point-of-care ultrasound examination of the lungs is a fast, cost-effective, non-invasive, and radiation-free method that can more easily, and with higher diagnostic accuracy, identify pneumothoraces.
